# *Hovenia dulcis Thunb*. Honey Exerts Antiviral Effect Against Influenza A Virus Infection Through Mitochondrial Stress-Mediated Enhancement of Innate Immunity

**DOI:** 10.3390/antiox14010071

**Published:** 2025-01-09

**Authors:** Eun-Bin Kwon, Buyun Kim, Young-Eun Kim, Sung-Joon Na, Sang Mi Han, Soon Ok Woo, Hong Min Choi, Siwon Moon, Young Soo Kim, Jang-Gi Choi

**Affiliations:** 1 Korean Medicine Application Center, Korea Institute of Oriental Medicine, Daegu 41062, Republic of Korea; wrld2931@kiom.re.kr (E.-B.K.); bykim@kiom.re.kr (B.K.); kye9912@naver.com (Y.-E.K.); 2 Special Forest Resources Division, National Institute of Forest Science, Suwon 16631, Republic of Korea; nsj10@forest.go.kr; 3 Department of Agricultural Biology, National Academy of Agricultural Science, Rural Development Administration, Wanju 566-851, Republic of Korea; sangmih@korea.kr (S.M.H.); wooso1@korea.kr (S.O.W.); sgiantm@korea.kr (H.M.C.); moonsiwon@korea.kr (S.M.)

**Keywords:** *Hovenia dulcis Thunb*., influenza A virus, cGAS–STING pathway, innate immune system, reactive oxygen species

## Abstract

To combat influenza A virus (IAV) infection, it is vital to develop effective therapeutic strategies, including immunomodulators. In this study, we examined the antiviral effects of Hovenia dulcis Thunb. honey (HDH) against IAV using RAW 264.7 cells. HDH treatment significantly reduced IAV infection and viral protein expression. Moreover, it enhanced the production of interferon (IFN)-β, activated the innate immune response through the cyclic GMP–AMP synthase (cGAS)–stimulator of interferon genes (STING) pathway, and upregulated IFN signaling through signal transducer and activator of transcription (STAT)1/2 phosphorylation and interferon-stimulated gene (ISG) expression. In addition, HDH decreased IAV-induced intracellular and mitochondrial reactive oxygen species (ROS) production by upregulating the expression of antioxidant proteins, such as Sirt3 and SOD2. The results suggest that HDH is a potential therapeutic agent inhibiting viral replication and boosting host antiviral immunity.

## 1. Introduction

Influenza viruses are a major global health problem, posing a serious threat to human populations through seasonal outbreaks and occasional pandemics. These infections contribute significantly to morbidity and mortality worldwide, particularly among vulnerable populations such as elderly people, children, and individuals with compromised immune systems. In addition to the health impact, influenza outbreaks place a substantial economic burden on healthcare systems and societies due to increased medical costs and productivity losses [1,2,3]. Vaccines and antiviral drugs provide some protection; however, their efficacy is often limited because the virus undergoes rapid mutations, leading to vaccine mismatches and antiviral resistance. Thus, there is a pressing need for new treatment strategies. One promising approach is the exploration of natural products with antiviral properties, particularly those that can enhance innate immune defenses [4,5,6].

Innate immunity is crucial in protecting the body from various infections, including viral infection. It serves as the body’s first line of defense, providing a rapid, nonspecific response to viral pathogens [7,8]. Viruses replicate rapidly, so the ability of innate immunity to elicit a response within hours of acquiring an infection is essential in controlling their spread before the viral load increases [9]. In macrophages, mitochondrial stress contributes to the release of damage-associated molecular patterns (DAMPs), such as mitochondrial DNA (mtDNA), which stimulate innate immune receptors and downstream pathways, thus implicating mitochondria as targets and initiators of the innate immune response.

mtDNA escapes into the cytoplasm and is recognized by the DNA sensor cyclic GMP–AMP synthase (cGAS), which activates stimulator of interferon genes (STING)–interferon regulatory factor 3 (IRF3)-dependent signaling, inducing interferon (IFN)-stimulated gene (ISG) expression [10,11,12,13]. This augments the type 1 IFN response and confers broad-spectrum viral resistance. In addition, ROS generated by mitochondrial stress activate innate immune pathways, including type I IFN induction and activation of immune cells, such as macrophages and natural killer cells [14,15,16]. This immediate response is necessary to limit the early stages of infection and prevent systemic infection before the adaptive immune system can elicit a more focused attack.

Recent studies have shown that honey exhibits antiviral activity against influenza A virus (IAV) through the enhanced innate immune system [17,18]. Therefore, we examined the antiviral effects exhibited by honey obtained from different sources, through innate immune activation. Of the honey types tested, *Hovenia dulcis Thunb*. honey (HDH), which is rich in minerals and antioxidants, is effective against arthritis and muscle pain. It also has hangover-relieving and liver-protecting effects. Moreover, honey is rich in polyphenols, flavonoids, and other bioactive compounds and has garnered increased attention for its potential to combat viral infections [19,20].

This study explores the hypothesis that HDH inhibits IAV infection through the mitochondrial stress-mediated activation of innate immunity. We elucidated the antiviral defense-enhancing role of HDH as an alternative approach to mitigating IAV infection by identifying the molecular pathways involved and their potential therapeutic implications.

## 2. Materials and Methods

### 2.1. HDH

HDH was provided by the Korea Beekeeping Cooperative (Namyangju, Gyeonggi-do, Republic of Korea) and Rural Development Administration (Jeonju, Jeollabuk-do, Republic of Korea).

### 2.2. Cells and Viruses

RAW 264.7 cells were obtained from the American Type Culture Collection (Manassas, VA, USA). The cells were maintained in Dulbecco’s modified Eagle medium (DMEM) containing 10% fetal bovine serum (FBS) and 1% penicillin–streptomycin at 37 °C in a 5% CO2 atmosphere. DMEM, FBS, and penicillin–streptomycin was purchased from Hyclone (Pittsburgh, PA, USA). Green fluorescent protein (GFP)-conjugated influenza A strains (A/PR8/34–GFP) and Puerto Rico/8/34 viruses were used as previously described [21].

### 2.3. Detection of Viral GFP

RAW 264.7 cells were treated with HDH (1.25, 2.5, and 10 mg/mL) or 1000 units of IFN-beta (IFN-β) as a positive control and incubated for 24 h before infection with A/PR/8/34–GFP (multiplicity of infection, MOI = 5). GFP levels were measured post-infection at 200× magnification using a fluorescence microscope (Nikon, Tokyo, Japan). Cells were harvested and subjected to flow cytometry (CytoFLEX, Beckman, Brea, CA, USA).

### 2.4. Detection of IFN-β

RAW 264.7 cells were incubated with varying concentrations of HDH (1 and 5 mg/mL) or 100 ng/mL of lipopolysaccharides (LPSs) as the positive control for 24 h. Supernatants were collected, and IFN-β (mouse) levels were measured using ELISA antibody kits following the manufacturer’s instructions (PBL Assay Science, Piscataway, NJ, USA). Intracellular IFN-β expression was confirmed using the IFN-β FACS antibody and HRP-conjugated IFN-β antibody by flow cytometry and Western blot analysis.

### 2.5. Detection of ROS

RAW 264.7 cells were treated with HDH (2.5 and 10 mg/mL) or 1000 units of IFN-beta (IFN-β) as a positive control and incubated for 24 h before infection with A/PR/8/34. After incubation, mitochondrial stress was assessed using fluorescent dyes, namely, dichlorodihy-drofluorescein diacetate (DCF-DA) and MitoSOX. All assays were performed according to the manufacturer’s protocols (Thermo Fisher Scientific, Waltham, MA, USA). Briefly, cells were treated with 2 μM DCF-DA or 1 μM MitoSOX and incubated for 30 min at 37 °C, after which fluorescence was measured using a flow cytometer.

### 2.6. Western Blot Analysis

Protein expression was analyzed by Western blot analysis with cell lysates prepared using PRO-PREP protein extraction solution (Intron Biotechnology, Seoul, Republic of Korea). The protein concentration was measured using the Bradford method, and the samples were separated by sodium dodecyl sulfate-polyacrylamide gel electrophoresis on 8–15% gels. Following transfer to polyvinylidene fluoride membranes, the membranes were blocked with 0.5 × Ez-Block Chemi (Amherst, MA, USA). The membranes were incubated with specific antibodies and visualized using the ChemiDoc imaging system (UVITEC, Cleaver Scientific Ltd., Cambridge, UK) and an enhanced chemiluminescence reagent (Thermo Scientific, Rockford, IL, USA). Protein band intensities were quantified using ImageJ Version 1.54 software.

### 2.7. Statistical Analysis

Data are expressed as the mean ± SEM. Significant differences in mean values between the treated and control groups were determined by one-way ANOVA. Tukey’s post hoc test was used for multigroup comparisons. Statistical analyses were performed using GraphPad PRISM^®^ Version 8.01 software (GraphPad, San Diego, CA, USA), and *p* < 0.05 was considered statistically significant.

## 3. Results

### 3.1. Antiviral Effect of HDH Against IAV in RAW 264.7 Cells

**Figure 1:**
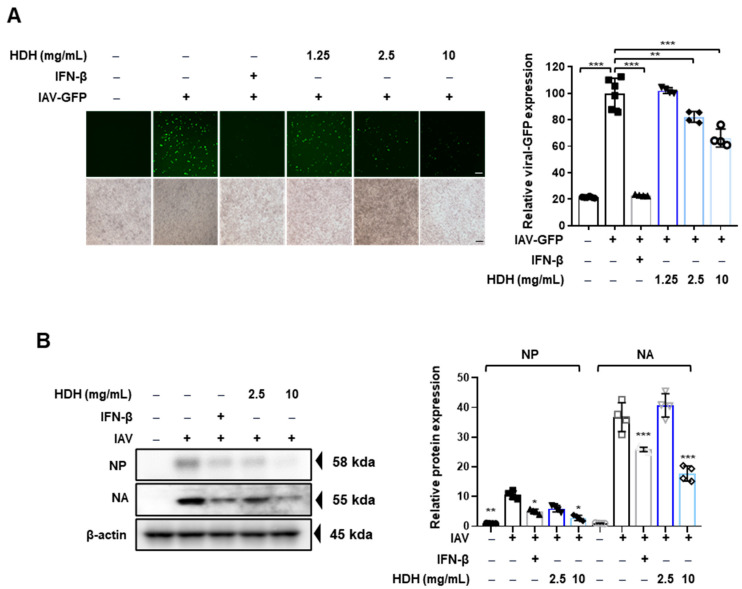
Antiviral effect of HDH in Raw264.7 cells. The cells were pre-treated with HDH at concentrations of 1.25, 2.5, and 10 mg/mL before infection with IAV-GFP. Green fluorescence represents infected cells. (**A**) Fluorescence images were captured under a microscope (left), and the percentage of infected cells was quantified by flow cytometry (right). Scale bar = 100 μm. (**B**) Viral protein levels (NP and NA) were assessed by Western blotting (left) and quantified using ImageJ software (right). The experiment was performed three times independently. Bar graph (mean ± SEM) statistics were determined using a one-way ANOVA with Tukey’s post hoc test; *** *p* < 0.001, ** *p* < 0.01, and * *p* < 0.05 compared with the virus infection group.

To investigate the anti-influenza A virus (IAV) effects of HDH, RAW 264.7 cells were treated with various concentrations of HDH (1.25, 2.5, and 10 mg/mL) for 24 h. Following this pre-treatment, the cells were infected with the A/PR8/34–GFP virus (multiplicity of infection [MOI] = 5), a strain of IAV engineered to express green fluorescent protein (GFP) as a marker of infection. As shown in Figure 1A, treatment with HDH led to a dose-dependent reduction in viral GFP expression, indicating a marked decrease in viral replication.

Further analysis was conducted using Western blotting to examine the impact of HDH on specific viral proteins. This analysis demonstrated that HDH significantly inhibited the expression of key IAV proteins, including nucleoprotein (NP) and neuraminidase (NA), in RAW 264.7 cells, as depicted in Figure 1B. These results collectively suggest that HDH exerts potent anti-IAV activity by inhibiting both viral infection and the expression of critical viral proteins in host cells.

### 3.2. HDH Enhances IFN-β Production

**Figure 2:**
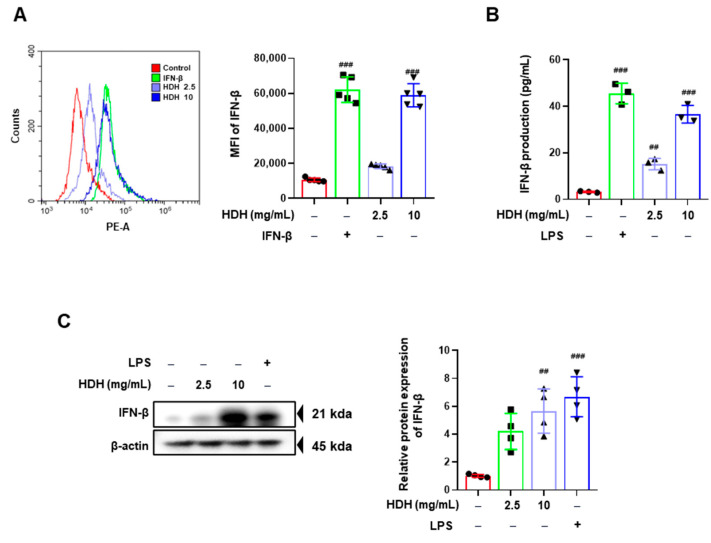
HDH enhanced the expression of IFN-β in Raw264.7 cells. The cells were treated with HDH at concentrations of 2.5 and 10 mg/mL for 24 h. (**A**) IFN-β expression levels were measured by intracellular staining followed by flow cytometry. (**B**) The IFN-β secretion was measured using an ELISA Kit. (**C**) Expression of IFN-β was assessed by Western blotting (left) and quantified using ImageJ software (right). The experiment was performed three times independently. Bar graph (mean ± SEM) statistics were determined using a one-way ANOVA with Tukey’s post hoc test; ### *p* < 0.001 and ## *p* < 0.01 compared with the untreated group.

Immune cytokines, including type I interferons like interferon-beta (IFN-β), play a crucial role in mediating the antiviral response of macrophages by enhancing their capacity to limit viral replication and spread [22,23]. To assess the impact of HDH on the antiviral immune response, we evaluated its effect on IFN-β secretion in RAW 264.7 macrophages. The intracellular expression of IFN-β was initially analyzed by flow cytometry using an antibody specific to IFN-β. The results demonstrated a significant increase in IFN-β expression within RAW 264.7 cells following HDH treatment (Figure 2A), suggesting that HDH enhanced the cellular antiviral response. To determine whether this increase in intracellular IFN-β also resulted in enhanced cytokine secretion, supernatants from HDH-treated RAW 264.7 cells were collected, and the amount of secreted IFN-β was quantified using an enzyme-linked immunosorbent assay (ELISA). We used LPS as a positive control because it robustly induces the production of inflammatory cytokines such as IFN-β and TNF-α, which are essential in studying macrophage activation and innate immune responses. As shown in Figure 2B, HDH and LPS-treated cells secreted significantly higher levels of IFN-β than untreated controls. To further confirm the increase in IFN-β expression, intracellular proteins were isolated and analyzed by Western blotting. This analysis corroborated the flow cytometry findings, revealing an elevated level of IFN-β in HDH-treated cells compared to the negative control group (Figure 2C).

In summary, these findings suggest that HDH stimulates the innate immune response in RAW 264.7 cells by inducing the production and secretion of critical antiviral cytokines, such as IFN-β. This upregulation of IFN-β expression and secretion highlights HDH’s potential role in enhancing the macrophage-mediated antiviral defense.

### 3.3. HDH Activates the Innate Immune Response

**Figure 3:**
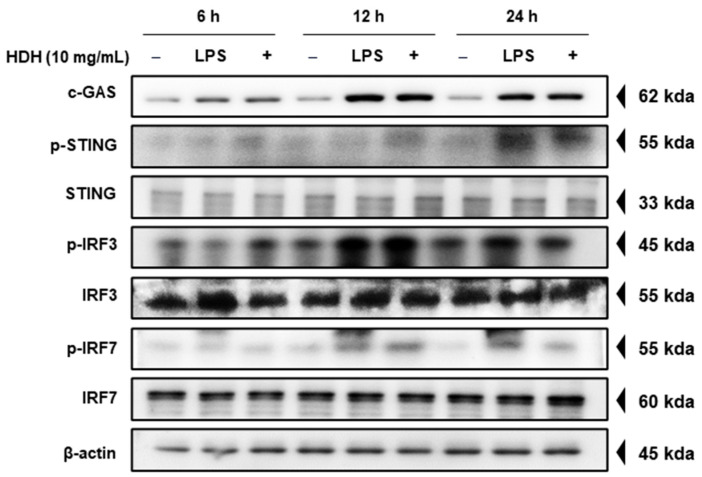
HDH activates the cGAS-STING pathway in Raw264.7 cells. The cells were treated with HDH at concentrations of 10 mg/mL for 6, 12, and 24 h. cGAS, STING, IRF3, and IRF7 were tracked by Western blotting.

To investigate whether HDH activates innate immune signaling pathways, we specifically examined components of the cyclic GMP-AMP synthase (cGAS)–stimulator of interferon genes (STING) pathway, as well as downstream factors like interferon regulatory factors 3 and 7 (IRF3/7), which are known to play critical roles in antiviral defense mechanisms [11]. Western blot analysis was conducted to measure the expression and phosphorylation status of these key signaling molecules following HDH treatment. As shown in Figure 3, HDH treatment led to a time-dependent increase in cGAS expression and STING phosphorylation, along with elevated levels of IRF3 and IRF7 phosphorylation, indicating that HDH activates these essential antiviral pathways in RAW 264.7 cells. This suggests a potential mechanism by which HDH could enhance innate immune defenses against viral infections through the activation of the cGAS-STING pathway and downstream signaling components

### 3.4. HDH Upregulates IFN Signaling Pathway

**Figure 4:**
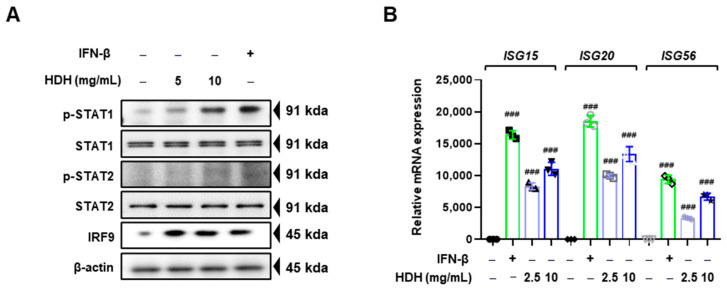
HDH increased the activity of the interferon signaling pathway. Raw264.7 cells were treated with HDH at concentrations of 2.5 and 10 mg/mL for 24 h. (**A**) The expression of STAT1, STAT2, and IRF9 was analyzed by Western blot after isolating proteins from the cells. (**B**) The expression of *ISG15, ISG20*, and *ISG56* were detected by RT-PCR. The experiment was performed three times independently. Bar graph (mean ± SEM) statistics were determined using a one-way ANOVA with Tukey’s post hoc test; ### *p* < 0.001 compared with the untreated group.

IFN-β drives the antiviral response cascade by inducing the activation of the transcription factor ISGF3 (a complex comprising phosphorylated STAT1, STAT2, and IRF9) [24]. We evaluated the effect of HDH on ISGF3 expression by Western blot analysis. The results indicated that HDH increased STAT1 and STAT2 phosphorylation and IRF3 expression (Figure 4A). In addition, ISGF3 activation induced ISG expression [25]. Therefore, we determined the effect of HDH on ISG expression via RT-PCR. As shown in Figure 4B, HDH upregulated the expression of ISGs, including *ISG15*, *ISG20*, and *ISG56*. To summarize, HDH induced the expression of antiviral ISG through the activation of STAT1/2 and IRF9.

### 3.5. HDH Reduces ROS in IAV-Infected Cells

**Figure 5:**
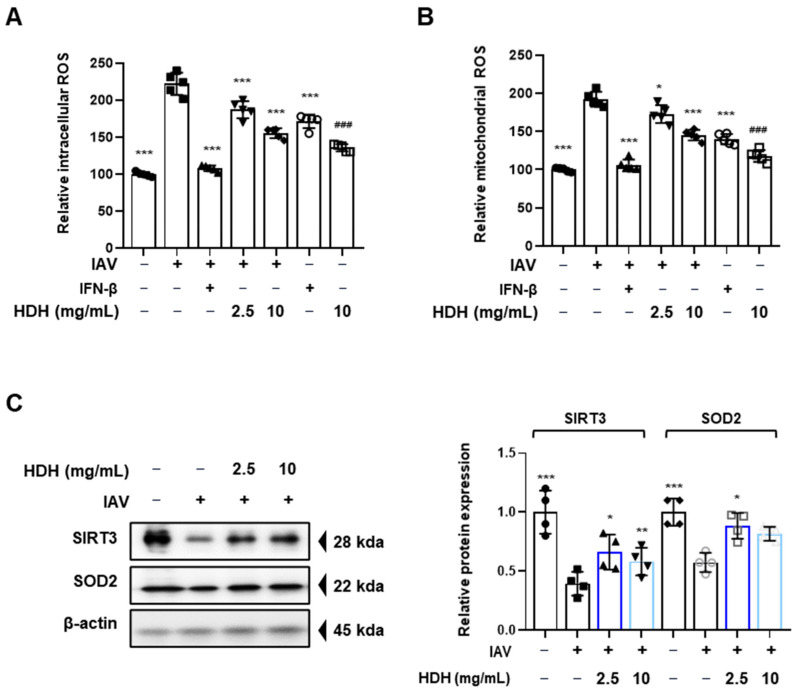
Antioxidant effect of HDH on IAV-infected Raw264.7 cells. The cells were pre-treated with HDH at concentrations of 2.5 and 10 mg/mL before infection with IAV. (**A**) Intracellular ROS were measured by DCF-DA, and (**B**) mitochondrial ROS were measured by staining by MitoSOX following by flow cytometry. (**C**). The expression of SIRT3 and SOD2 were assessed by Western blotting (left) and quantified using ImageJ software (right). The experiment was performed three times independently. Bar graph (mean ± SEM) statistics were determined using a one-way ANOVA with Tukey’s post hoc test; *** *p* < 0.001, ** *p* < 0.01, and * *p* < 0.05 compared with the virus infection group. ### *p* < 0.001 compared with the untreated group.

IAV increases ROS production, which suppresses the antiviral response and contributes to pathological inflammation and morbidity in inflammatory cells, including macrophages, monocytes, and epithelial cells [26]. To determine the effect on intracellular and mitochondrial ROS inhibition by HDH treatment for IAV infection, we measured ROS levels by flow cytometry. To determine the extent of intracellular and mitochondrial ROS induction by IAV infection, cells were treated with HDH before IAV infection, and ROS levels were measured 24 h post-infection. As shown in Figure 5A,B, the virus elevated intracellular and mitochondrial ROS; however, HDH treatment reduced intracellular and mitochondrial ROS. Sirt3 controls mitochondrial oxidative signaling and regulates mitochondrial proteins, such as SOD2, to improve the antioxidant defense system [27]. We evaluated the effect of HDH on Sirt3 and SOD2 expression by Western blot analysis. The results indicated that the virus downregulated Sirt3 and SOD2 expression; however, HDH upregulated Sirt3 and SOD2 expression in IAV-infected cells (Figure 5C). To summarize, HDH ameliorates the antioxidant system induced by viral infection by activating the antioxidant proteins Sirt3 and SOD2.

## 4. Discussion

The findings of this study demonstrate that HDH exerts a potent antiviral effect against IAV through the activation of the innate immune response, specifically by enhancing type I IFN production and reducing ROS production. This study contributes to our understanding of how HDH can serve as a natural antiviral agent by targeting key innate immune pathways, which offers a complementary approach to traditional antiviral therapies. The results indicate that HDH treatment significantly reduces the expression of IAV proteins (NP and PB1) and viral GFP expression in infected RAW 264.7 cells, suggesting that HDH exerts direct antiviral effects by interfering with the replication and expression of viral genes. The ability of HDH to inhibit viral replication is consistent with previous studies highlighting the antiviral properties of various honey types, supporting the idea that the bioactive compounds in honey can effectively target viral infections.

A key aspect of the antiviral mechanism of HDH is its ability to enhance intracellular IFN-β production in RAW 264.7 cells, as confirmed by flow cytometry, Western blot analysis, and ELISA. Type I IFNs, such as IFN-β, play an important role in the early antiviral response by initiating ISG expression, which can inhibit viral replication and spread. The enhanced IFN-β production in response to HDH treatment underscores its potential role in boosting the innate immune response following IAV infection. Further studies into the underlying mechanisms have revealed that HDH activates the cGAS–STING pathway, which is an important mediator of DNA sensing in innate immunity. The activation of STING and subsequent phosphorylation of IRF3/7 following HDH treatment suggests that it promotes the release of mtDNA into the cytoplasm, which triggers the cGAS–STING signaling cascade. This pathway is essential in inducing the production of IFN-β and ISGs, thereby enhancing antiviral defense. The results are consistent with the known role of mtDNA as a damage-associated molecular pattern (DAMP) that activates innate immune receptors in response to viral infection. In addition to activating IFN-β signaling, HDH also induced the phosphorylation of STAT1 and STAT2, which are key components of the ISGF3 complex. This upregulated the expression of ISGs, such as ISG15, ISG20, and ISG56, which inhibit various stages of the viral life cycle [28,29]. The upregulation of these antiviral genes further supports the role of HDH in amplifying the antiviral state of macrophages, which offers a robust defense against IAV.

HDH also mitigated the IAV-induced increase in ROS levels in infected cells. Viral infections, including those caused by IAV, induce oxidative stress, which can suppress the antiviral response and exacerbate inflammation [30]. By reducing intracellular and mitochondrial ROS levels, HDH maintains cell homeostasis and prevents excessive oxidative damage. The upregulation of antioxidant proteins, such as Sirt3 and SOD2, suggests that HDH enhances the antioxidant defense system in IAV-infected cells. This ability to modulate oxidative stress may provide a dual benefit by supporting innate immune activation while reducing virus-induced inflammatory damage [31]. The interplay between ROS reduction and the activation of antiviral pathways highlights a unique feature of HDH. Although ROS are typically associated with activating the innate immune response, excessive ROS levels can be detrimental. HDH appears to balance ROS levels, enabling effective immune activation without triggering excessive inflammation. This balance may be particularly advantageous in controlling the inflammatory response during viral infection, suggesting that HDH is a useful treatment for mitigating viral replication and its associated pathologies.

In this study, RAW 264.7 cells were used as a model to investigate the innate immune-modulating effects of HDH, particularly its activation of the cGAS-STING pathway. While this cell line provides a robust and reproducible system to study macrophage-mediated antiviral responses, it is limited by its murine origin, which may not fully replicate human immune responses. Furthermore, the in vitro nature of these experiments does not account for the complexities of systemic immune regulation and interactions in vivo. Human cell lines such as THP-1, A549, or Calu-3 offer advantages in mimicking in vivo conditions, and future studies should focus on validating these findings in such models. Additionally, animal models and human tissue cultures could provide valuable insights into the broader applicability of HDH as a therapeutic agent and its potential efficacy in treating respiratory viral infections. Moreover, future research should also prioritize the detailed compositional analysis of HDH to identify the specific bioactive compounds responsible for its antiviral effects. Evaluating the individual and synergistic effects of these components will make it possible to better understand their mechanisms of action and optimize the therapeutic potential of HDH. These efforts will help to bridge the gap between in vitro findings and practical applications in antiviral therapy.

**Figure 6:**
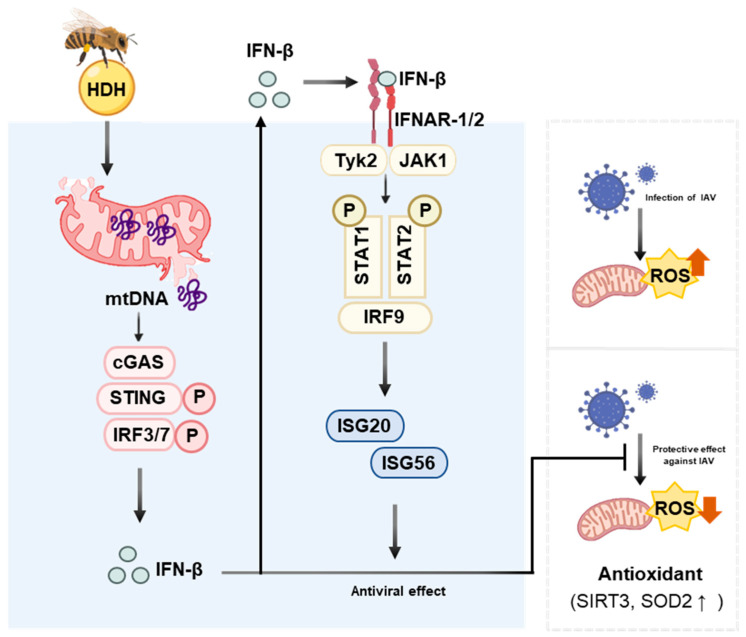
Protective effect of HDH against IAV via activation of the innate immune system. HDH enhances the secretion of IFN-β though the cGAS-STING pathway. Additionally, HDH suppresses viral infection by increasing antiviral signaling pathways and reduces reactive oxygen species levels increased by viral infection.

Collectively, these findings suggest that HDH acts through multiple mechanisms to counteract IAV infection, including the direct inhibition of viral replication, the enhancement of type I IFN production, the activation of innate immune pathways, the upregulation of antiviral genes, and the modulation of the host antioxidative defense system (Figure 6). This multifaceted approach highlights the potential of HDH as a natural treatment for IAV. In addition, investigating the broader antiviral spectrum of HDH against other respiratory viruses would provide valuable insights into its potential applications in treating viral infections beyond IAV.

## 5. Conclusions

This study demonstrated that HDH inhibits IAV infection in RAW 264.7 cells by reducing viral protein expression, increasing IFN-β and ISG production, activating innate immune pathways, and reducing the ROS level through the upregulation of Sirt3 and SOD2. Therefore, HDH may be an effective therapeutic strategy, in combination with immunomodulators, to combat IAV infection.

## Data Availability

All data are available within the article.
